# SIK2 enhances synthesis of fatty acid and cholesterol in ovarian cancer cells and tumor growth through PI3K/Akt signaling pathway

**DOI:** 10.1038/s41419-019-2221-x

**Published:** 2020-01-13

**Authors:** Jing Zhao, Xiaohong Zhang, Tian Gao, Shanci Wang, Yiran Hou, Peng Yuan, Yi Yang, Tao Yang, Jinliang Xing, Jibin Li, Shujuan Liu

**Affiliations:** 1 State Key Laboratory of Cancer Biology and Department of Physiology and Pathophysiology, Fourth Military Medical University, Xi’an, Shaanxi 710032 China; 2 Department of Gynaecology and Obstetrics, Xijing Hospital, Fourth Military Medical University, Xi’an, Shaanxi 710032 China; 3 Guangdong Provincial Key Laboratory of Gastroenterology, Department of Gastorenterology, Nanfang Hospital, Southern Medical University, Guangzhou, China; 4 Medical College of Yan’an University, Yan’an, Shaanxi 716000 China; 5 Department of Pain Treatment, Tangdu Hospital, The Fourth Military Medical University, Xi’an, Shaanxi 710038 China; 6 State Key Laboratory of Cancer Biology and Experimental Teaching Center of Basic Medicine, Fourth Military Medical University, Xi’an, Shaanxi 710032 China

**Keywords:** Cancer metabolism, Cancer therapy, Oncogenes

## Abstract

Salt-inducible kinase 2 (SIK2) has been established as a regulator of diverse biological processes including cell metabolism. A recent study has reported that SIK2 is required for adipocyte-induced ovarian cancer (OC) survival through facilitating fatty acid oxidation. However, whether SIK2 also plays a role in the lipid synthesis in OC cells remains elusive. Here, we showed that SIK2 significantly promoted the lipid synthesis in OC cells. On the one hand, SIK2 enhanced fatty acid synthesis through upregulating the expression of sterol regulatory element binding protein 1c (SREBP1c) and thus the transcription of major lipogenic enzyme FASN. On the other hand, SIK2 promoted cholesterol synthesis through upregulating the expression of sterol regulatory element binding protein 2 (SREBP2) and thus the transcription of major cholesterol synthesis enzymes HMGCR. Moreover, PI3K/Akt signaling pathway was found to be involved in the upregulation of SREBP1c and SREBP2 in OC cells. Moreover, in vitro and in vivo assays indicated that the SIK2-regulated fatty acid and cholesterol synthesis played a critical role in the growth of OC cells. Our findings demonstrate that SIK2 is a critical regulator of lipid synthesis in OC cells and thus promotes OC growth, which provides a strong line of evidence for this molecule to be used as a therapeutic target in the treatment of this malignancy.

## Introduction

Dysregulation of fatty acid metabolism has been increasingly recognized as a component of malignant transformation in many different cancers, including ovarian cancer^1,2^. Elevated de novo fatty acid synthesis provides cancer cells with building blocks, signaling molecules and post-translational modifications to promote rapid cell proliferation. To date, many enzymes which are involved in de novo fatty acid biosynthesis, such as ATP citrate lyase (ACLY), acetyl-CoA carboxylase (ACC), fatty acid synthase (FASN) and stearoyl-CoA desaturase1 (SCD1), are overexpressed and contributed to poor clinical outcomes in many different types of cancers^3,4^. Compared with most current studies focusing on de novo fatty acid synthesis, the functional roles of cholesterol in cancer development has received less attention^5,6^. Hypercholesterolemia has been considered as an important risk factor for cancers^7,8^. Except for serum cholesterol, intracellular cholesterol also has been shown to play a crucial role in the regulation of tumor progression. Elevation of intracellular cholesterol level has been observed in tumor tissues^9,10^, which promoted the proliferation, migration and invasion of cancer cells. Besides, several recent studies also have demonstrated that the increased expression of cholesterol synthesis genes is associated with the decreased patient survival. Chushi Li et al. have reported that 3-hydroxy-3-methyl-glutaryl-coenzyme A reductase (HMGCR), the rate-limiting enzyme for cholesterol synthesis is up-regulated in gastric cancer and positively regulates the growth and migration of cancer cells^11^. The oncogenic roles of HMGCR have also been revealed in glioblastoma and esophageal squamous cell carcinoma^12,13^. Recently, there has been a revival of enthusiasm amongst investigators to study how lipid metabolism pathways are reprogrammed in cancer cells. However, mechanisms underlying the increased de novo fatty acid and cholesterol synthesis in cancer cells are still not completely understood and further study is still required.

Salt-inducible kinase 2 (SIK2) is an AMP-activated protein kinase (AMPK)-related protein kinase that plays important roles in the regulation of cellular metabolism. Besides, several studies have reported that SIK2 activates a series of signaling pathways, such as PI3K/Akt, Hippo-YAP and LKB1-HDAC, which are associated with diverse cellular processes^14^. Recent studies have unraveled the role of SIK2 in cancer development and progression. It has shown that SIK2 is required for the proliferation of both prostate cancer and ovarian cancer cells^15,16^. Moreover, a recent study has reported that SIK2 is highly expressed in adipocyte-rich metastases and required for adipocyte-induced proliferation of metastatic ovarian cancer through facilitating fatty acid oxidation^17^, implying that SIK2 may play a critical role in the regulation of fatty acid metabolism. However, the role of SIK2 in the regulation of lipid synthesis in cancer cells, especially in ovarian cancer (OC) cells, is still unclear.

In this study, we explored the functional role and the underlying molecular mechanisms of SIK2 in the regulation of lipid synthesis, including fatty acid and cholesterol synthesis, in OC cells.

## Materials and methods

### Antibodies and reagents

The primary antibodies used in this study and their working concentration were listed in Supplementary Table S1. The PI3K inhibitor LY294002 (Cat. no. HY-10108), SIK inhibitor HG-9-91-01 (Cat. no. HY-15776), FASN inhibitor C75 (Cat. no. HY-12364) and HMGCR inhibitor Mevastatin (Cat. no. HY-17408) were purchased from MedChemExpress (New Jersey, USA).

### Cell lines and tissue samples

Human OC cell lines A2780, HEY, SKOV3 and ES2 were obtained from the American Type Culture Collection (ATCC) and cultured in recommended Dulbecco’s Modified Eagle Medium (DMEM) and RPMI-1640 medium supplemented with 10% FBS, respectively. In addition, tumor tissues and matched peritumor tissues were obtained from 121 OC patients from the Department of Gynaecology and Obstetrics at Xijing Hospital between 2011 and 2015. The study has been approved by the Ethics Committee of Xijing Hospital and all patients provided written informed consent.

### Knockdown and overexpression of target genes

Small interference RNA (siRNA) targeting SIK2, SREBP1c, SREBP2 and control siRNA (Genepharma, Shanghai, China) were transfected into OC cell lines using the transfection reagent lipofectamine 2000 (Thermo Fisher Scientific, MA, USA) according to the manufacturer’s protocols. Sequences of siRNA used in the study were listed in Supplementary Table S2.

SIK2 overexpression vector and empty vector were kind gifts from Dr. Ahmed Ashour Ahmed (University of Oxford, UK). SREBP1c knockdown (shSREBP1c) and overexpression vectors have been constructed in our previous study^18^. SREBP2 overexpression vector were purchased from (Genepharma, Shanghai, China).

### Quantitative real-time PCR analysis

Total RNA was extracted from OC cells using RNA extraction Kit (TaKaRa, 9767, Japan) following the manufacturer’s instructions. Reverse transcription for complementary DNA (cDNA) synthesis was performed using Transcriptor Reverse Transcriptase (TaKaRa, RR036A, Japan). Quantitative real-time PCR (qRT-PCR) was used to determine the gene expression level using 2 × SYBR Green qPCR Master Mix (S2014, Everbright USA) with the primers listed in Supplementary Table S2. β-actin was used as an internal control.

### Western blot analysis

Proteins extracted from lysed OC cells were run on SDS-PAGE for separation and then transferred onto PVDF membranes followed by incubation with primary and HRP-conjugated second antibody antibodies as recommended by the manufacturer. Antibodies and their dilutions were listed in Supplementary Table S1 and β-actin served as an internal control. Quantification of the blots was done using Image J software.

### Quantification of free fatty acid, triglyceride, phospholipids and cholesterol

Lipids were extracted from OC cell homogenates. OC cells homogenates were prepared for lipids extraction using chloroform/methanol (2:1) and then quantitatively estimated for the levels of free fatty acid, triglycerideand, phospholipids and cholesterol with EnzyChromTM free fatty acid, triglyceride, phospholipidassay and cholesterol kits (Bioassay Systems, Hayward, CA, USA), following their manufacturers’ protocols.

### Quantification of neutral lipid

The content of neutral lipids in OC cells was monitored with a fluorescence dye BODIPY 493/503 (Invitrogen) following the manufacturer’s instruction. Briefly, after fixed with 4% paraformaldehyde, OC cells were then stained with BODIPY 493/503 (1 μg/mL) for 30 min at 37 °C. DAPI (20 μL/mL) was used for nuclei counterstain for 15 min. Confocal images were acquired with an Olympus FV-1000 confocal microscope (Olympus, Tokyo, Japan). Quantification of LDs was done using Image J software (Average numbers of LDs per cell, percentage of cellular area occupied by LDs).

### Immunohistochemical staining

Immunohistochemical (IHC) analysis was performed as previously described^19^. Briefly, 3-µm thick tissue sections were deparaffinized and hydrated. Hot citrate buffer (pH = 6.0) was used for antigen retrieval. Antigen was retrieved by treating with under pressure. The sections were then incubated with primary antibodies overnight at 4 °C. Color was developed using DAB and sections were then counterstained with hematoxylin. The immunostaining results were independently scored by two pathologists according to the intensity of staining as previously described^20^. The primary antibodies used in the study were listed in Supplementary Table S1.

### MTS cell viability and colony formation assay

Cell proliferation was measured using the CellTiter 96 Aqueous One Solution cell proliferation assay (Promega Corp, Madison, WI) following the manufacturer’s instructions. Briefly, OC cells were seeded into 96-well plates and incubated for 5 days. Cell proliferation was detected daily based on the absorbance at 490 nm using a microplate reader. For colony formation assay, 1000 OC cells were seeded into 6-well plate and cultured about 2 weeks. The colonies were stained with crystal violet and counted.

### EDU assay

A 5-ethynyl-2′-deoxyuridine (EdU)-incorporation assay kit (Ribobio, Guangdong, China) was used to measure the proliferation ability of OC cells following the manufacturer’s protocol.

### Wound-healing and transwell invasion assays

Wound-healing assay was performed to assess the migration of OC cells following the procedures as described previously^18^. For determination of OC cell invasion, a 6.5-mm Transwell chamber (Corning Costar, China) was used. Briefly, OC cells were collected and added into the top chamber of 24-well transwell plates. Simultaneously, cell culture medium containing 20% FBS were added into the bottom of transwell chamber. After culture for 48 h, invaded cells were stained with crystal violet and counted under a microscopy. The average number of inserted cells in five randomly selected fields was calculated.

### In vivo tumor growth assay

Six-week-old athymic female BALB/c nude mice were randomly divided into groups (six mice/group). A total of 1 × 10^7^ OC cells were injected subcutaneously into nude mice and the tumor size was assessed each week. The mice were sacrificed 40 days after injection and tumors were harvested and tumor size was compared between groups. All animal experiments were approved by the Institutional Animal Experiment Committee of Xijing Hospital.

### Statistical analysis

All values are presented as mean ± S.E.M from three independent experiments. Statistical significance was determined by one-way ANOVA or unpaired two-tailed Student’s *t*-test. *P*-value <0.05 was considered statistically significant. Correlations between measured variables were analyzed by Spearman rank correlation test.

## Results

### SIK2 significantly increased lipid contents in ovarian cancer cells

**Fig. 1:**
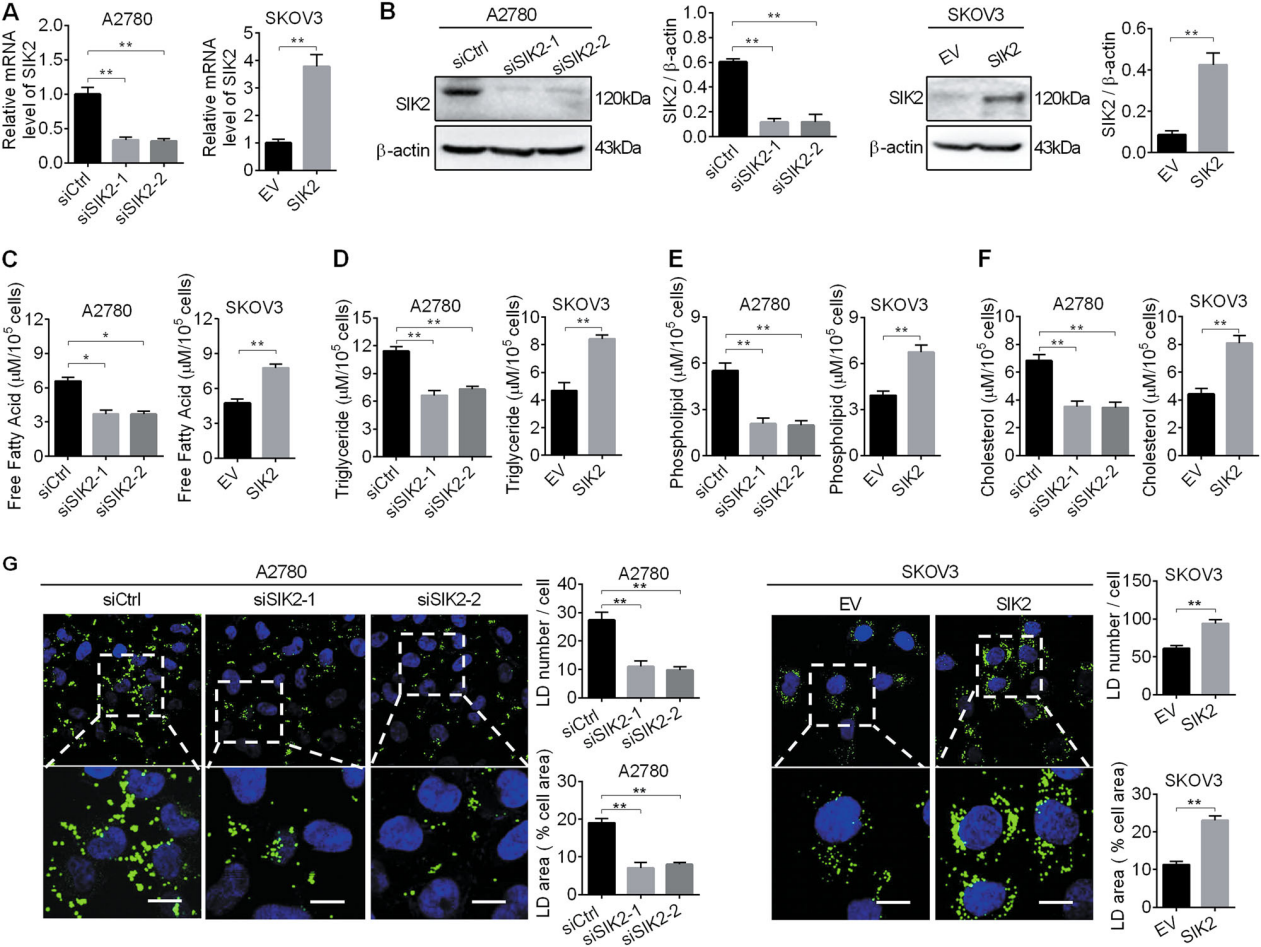
SIK2 significantly increased lipid contents in ovarian cancer (OC) cells. **a**, **b** Quantitative real-time PCR (**a**) and Western blot (**b**) analyses of SIK2 expression in ovarian cancer A2780 and SKOV3 cells with treatment as indicated. siSIK2–1 and siSIK2–2, siRNAs against SIK2; siCtrl, control siRNA; SIK2, expression vector encoding SIK2; EV, empty vector. **c**–**f** Cellular content of free fatty acid (**c**), triglyceride (**d**), phospholipids (**e**) and cholesterol (**f**) were detected in A2780 and SKOV3 cells with treatment as indicated. **g** The content of neutral lipids were detected by BODIPY 493/503 dye and counterstained with DAPI in A2780 and SKOV3 cells with treatment as indicated. Scale bars, 50 μm. Average numbers of LDs per cell (upper panel), percentage of cellular area occupied by LDs (lower panel). Data are shown as mean ± S.E.M from three independent experiments. *p < 0.05; **p < 0.01.

To investigate the potential role of SIK2 in the regulation of aberrant lipid metabolism in OC cells, the effect of SIK2 knockdown and overexpression on lipid content in A2780 cells with relative high SIK2 expression at both mRNA and protein levels and SKOV3 cells with relative low SIK2 expression at both mRNA and protein levels (Supplementary Fig. S1A, B) were firstly evaluated. The successful knockdown and overexpression of SIK2 was verified by qRT-PCR and Western blot analysis (Fig. 1a, b). Our data showed that A2780 cells with SIK2 knockdown had significantly decreased levels of intracellular free fatty acid, triglyceride, phospholipids and cholesterol when compared with control cells (Fig. 1c–f). In contrast, overexpression of SIK2 in SKOV3 cells increased the levels of intracellular free fatty acid, triglyceride, phospholipids and cholesterol. Keeping with the findings, cellular staining with the lipophilic dye BODIPY 493/503 provided further support, showing that SIK2 increased the intracellular contents of neutral lipids in OC cells (Fig. 1g). Collectively, our findings indicate that SIK2 promotes the reprogramming of lipid metabolism in OC cells.

### SIK2 upregulated the expressions of lipogenic enzymes in OC cells

**Fig. 2:**
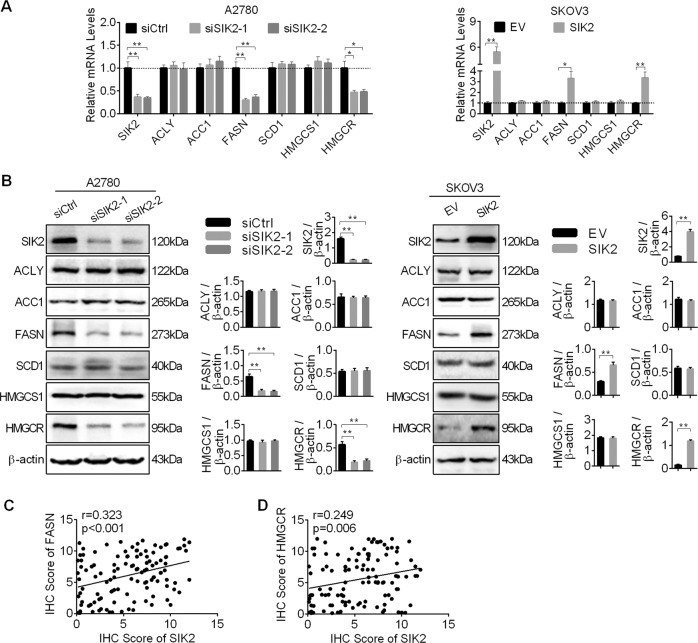
SIK2 upregulated the expressions of lipogenic enzymes in OC cells. **a** Quantitative RT-PCR analysis for mRNA expression levels of lipogenic enzymes ACLY, ACC1, FASN, SCD1, HMGCS1 and HMGCR in A2780 and SKOV3 cells with treatment as indicated. **b** Western blot analysis for protein expression levels of lipogenic enzymes of ACLY, ACC1, FASN, SCD1, HMGCS1 and HMGCR in A2780 and SKOV3 cells with treatment as indicated. **c**, **d** Spearman correlation analysis of the relationship between the protein expression levels of SIK2 and FASN, as well as between SIK2 and HMGCR based on IHC staining results. Data are shown as mean ± S.E.M from three independent experiments. *p < 0.05; **p < 0.01.

To study the role of SIK2 in the regulation of de novo fatty acid synthesis and cholesterol synthesis, we analyzed the expression of key enzymes involved in both fatty acid (ACLY, ACC1, FASN, SCD1) and cholesterol synthesis (HMGCS1, HMGCR). Our results showed that the expression levels of FASN and HMGCR were significantly decreased at both mRNA and protein levels in A2780 cells with SIK2 knockdown when compared with control cells, while SIK2 overexpression increased the expressions of FASN and HMGCR in SKOV3 cells (Fig. 2a, b). However, no significant changes of the expressions of ACLY, ACC1, SCD1 and HMGCS1 were observed when SIK2 was knockdown or overexpressed in A2780 and SKOV3 cells. To provide further support, the expression of SIK2 and FASN were determined by IHC analysis in tumor tissue samples from 121 OC patients. Spearman rank correlation analysis indicated a significant positive correlation of IHC scores between SIK2 and FASN (*r* = 0.323, *p* < 0.001) (Fig. 2c and Supplementary Fig. S2A), as well as between SIK2 and HMGCR (*r* = 0.249, *p* = 0.006) (Fig. 2d and Supplementary Fig. S2A).

### SIK2 upregulated FASN expression to promote fatty acid synthesis via SREBP1c

**Fig. 3:**
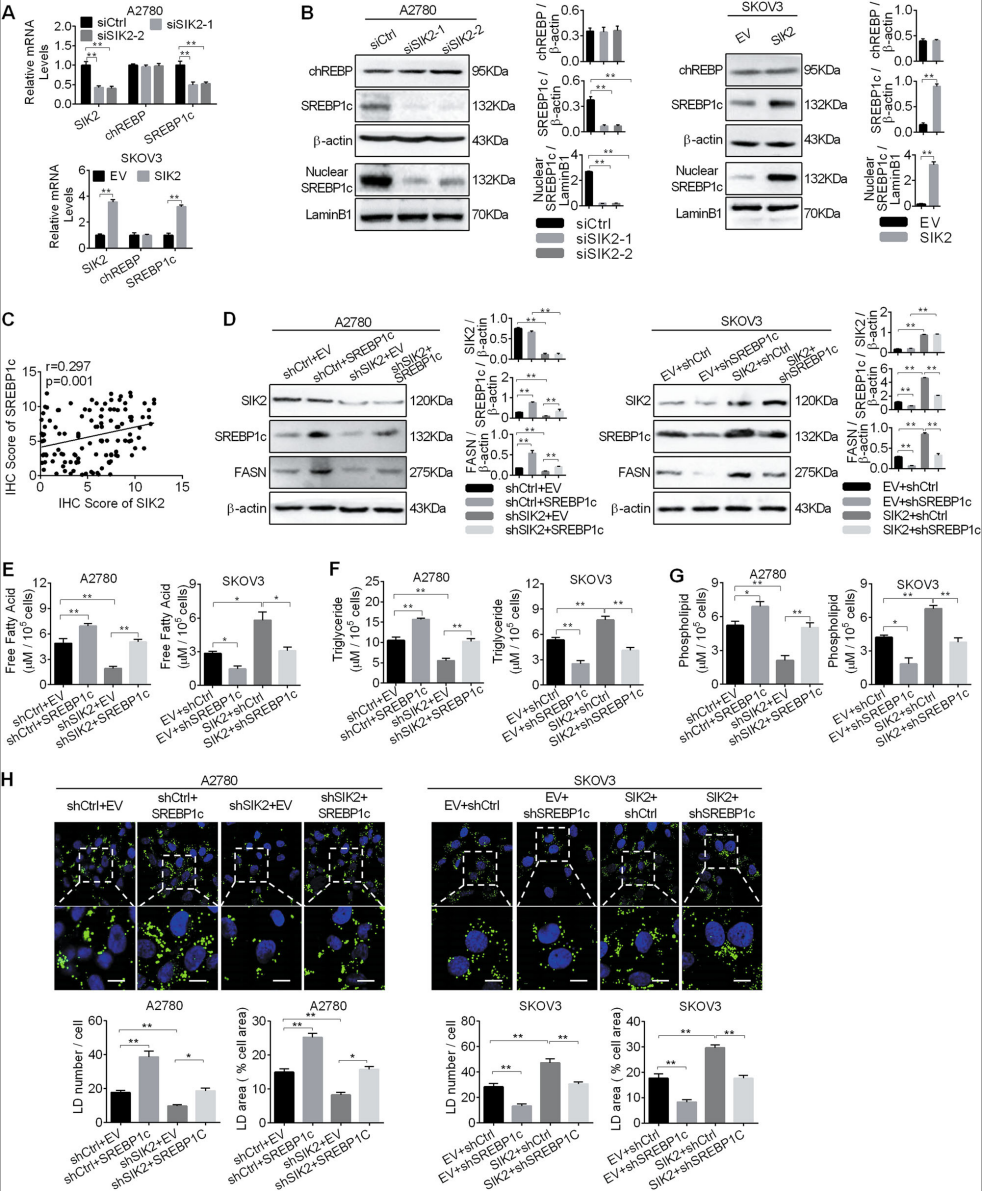
SIK2 upregulated FASN expression to promote fatty acid synthesis via SREBP1c. **a**, **b** Quantitative RT-PCR (**a**) and Western blot (**b**) analyses for expressions of chREBP and SREBP1c in A2780 and SKOV3 cells with treatment as indicated. **c** Spearman correlation analysis of the relationships between the protein expression levels of SIK2 and SREBP1c based on the IHC staining results. **d** Western blot analysis of SREBP1c and FASN in A2780 and SKOV3 cells with treatment as indicated (shSIK2, shRNA against SIK2; shCtrl, control shRNA; SREBP1c, expression vector encoding SREBP1c; EV, empty vector). **e–g** Cellular content of free fatty acid (**e**), triglyceride (**f**) and phospholipids (**g**) were detected in A2780 and SKOV3 cells with treatment as indicated. **h** The content of neutral lipids were detected by BODIPY 493/503 dye and counterstained with DAPI in A2780 and SKOV3 cells with treatment as indicated. Scale bars, 50 μm. Average numbers of LDs per cell (left), percentage of cellular area occupied by LDs (right). Data are shown as mean ± S.E.M from three independent experiments. *p < 0.05; **p *<* 0.01.

Carbohydrate-responsive element-binding protein (chREBP) and sterol regulatory elementary binding protein1c (SREBP1c) are important transcription factors in the regulation of fatty acid synthesis through transcriptional activation of lipogenic enzymes^21^. To explore the molecular mechanism of SIK2-mediated upregulation of lipogenic enzymes, the expression levels of chREBP and SREBP1c (the dominant isoform of SREBP1 in the ovary tissue^22^) in A2780 and SKOV3 cells were determined by qRT-PCR and Western blot analyses (Fig. 3a, b). Our results showed that only SREBP1c was significantly decreased at both mRNA and protein levels when SIK2 was knocked-down in A2780 cells, while no significant change was seen on the expression of chREBP. In contrast, SIK2 overexpression in SKOV3 cells increased SREBP1c expression. In addition, the level of nuclear SREBP1c, which represents the transcriptional activity of this molecule, showed a similar expression pattern to that of total SREBP1c, implying that SIK2 promoted the transcriptional activity of SREBP1c in OC cells. To provide further supports, the levels of SREBP1c was examined in tumor tissues from 121 OC patients. Spearman rank correlation analysis showed a positive correlation of expression levels between SIK2 and SREBP1c (*r* = 0.297, *p* = 0.001) (Fig. 3c and Supplementary Fig. S2A).

We further explored whether SREBP1c was involved in SIK2-mediated upregulation of FASN in OC cells. Our results showed that SIK2 knockdown significantly reduced the expressions of FASN, the contents of intracellular free fatty acid, triglyceride and phospholipids, as well as the intensity of BODIPY staining in A2780 cells, whereas overexpression of SREBP1c significantly restored the reduction. In contrast, the expression of FASN, content of intracellular free fatty acid, triglyceride and phospholipids, and intensity of BODIPY staining were clearly increased when SIK2 was overexpressed in SKOV3 cells (Fig. 3d–h). Expectedly, silencing of SREBP1c remarkably suppressed the lipogenisis-promoting effect of SIK2 overexpression.

### SIK2 upregulated HMGCR expression to promote cholesterol synthesis via SREBP2

**Fig. 4:**
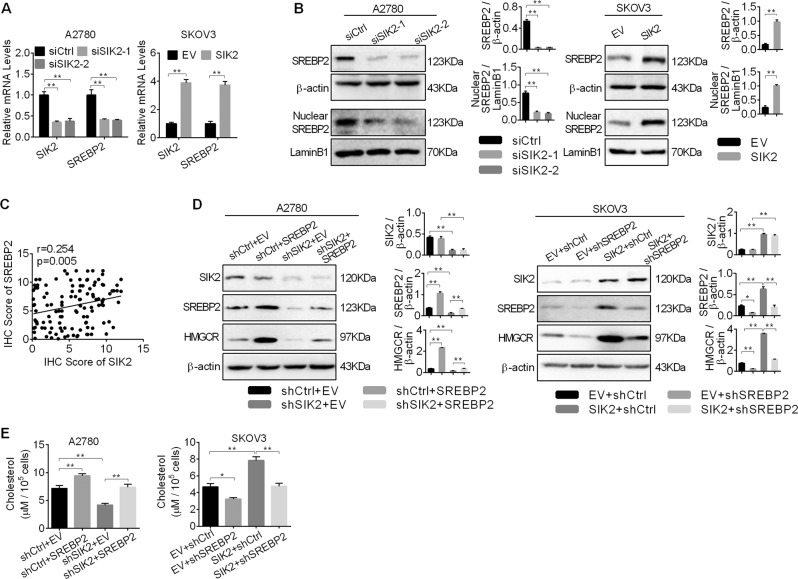
SIK2 upregulated HMGCR to promote cholesterol synthesis via SREBP2. **a**, **b** Quantitative RT-PCR (**a**) and Western blot (**b**) analyses for expressions of SREBP2 in A2780 and SKOV3 cells with treatment as indicated. **c** Spearman correlation analysis of the relationships between the protein expression levels of SIK2 and SREBP2 based on the IHC staining results. **d** Western blot analysis of SREBP2 and HMGCR in A2780 and SKOV3 cells with treatment as indicated (shSIK2, shRNA against SIK2; shCtrl, control shRNA; SREBP2, expression vector encoding SREBP2; EV, empty vector). **e** The intracellular levels of cholesterol was detected in A2780 and SKOV3 cells with treatment as indicated. Data are shown as mean ± S.E.M from three independent experiments. *p < 0.05; **p *<* 0.01.

Next, we explored the molecular mechanism of SIK2-mediated upregulation of cholesterol synthesis enzyme HMGCR. Considering that SREBP2 is well-known as a key regulator of cholesterol synthesis through transactivation of genes involved in cholesterol biosynthesis, the effect of SIK2 knockdown on the expression of SREBP2 was examined by qRT-PCR and Western blot analyses. As shown in Fig. 4a, b, SREBP2 was significantly decreased at both mRNA and protein levels when SIK2 was knocked-down in A2780 cells, whereas SIK2 overexpression significantly increased SREBP2 expression in SKOV3 cells. In addition, the nuclear expression of SREBP2, which represents the transcriptional activity of this molecule, showed a similar expression pattern to that of total SREBP2 (Fig. 4b), implying that SIK2 promotes the transcriptional activity of SREBP2 in OC cells. Moreover, spearman rank correlation analysis also showed a positive correlation of expression levels between SIK2 and SREBP2 in tumor tissues from 121 OC patients (*r* = 0.254, *p* = 0.005) (Fig. 4c and Supplementary Fig. S2A).

We further explored whether SREBP2 was involved in SIK2-upregulated HMGCR and cholesterol synthesis in OC cells. Our results showed that SIK2 knockdown significantly reduced the expression of HMGCR and the intracellular levels of cholesterol, whereas overexpression of SREBP2 significantly restored the reduction (Fig. 4d, e). In contrast, the expression of HMGCR and the intracellular levels of cholesterol was clearly increased when SIK2 was overexpressed in SKOV3 cells, whereas silencing of SREBP2 remarkably suppressed the intracellular levels of cholesterol of SIK2 overexpression (Fig. 4d, e).

### SIK2 promoted the upregulation of SREBP1c and SREBP2 by activating the PI3K/Akt signaling pathway

**Fig. 5:**
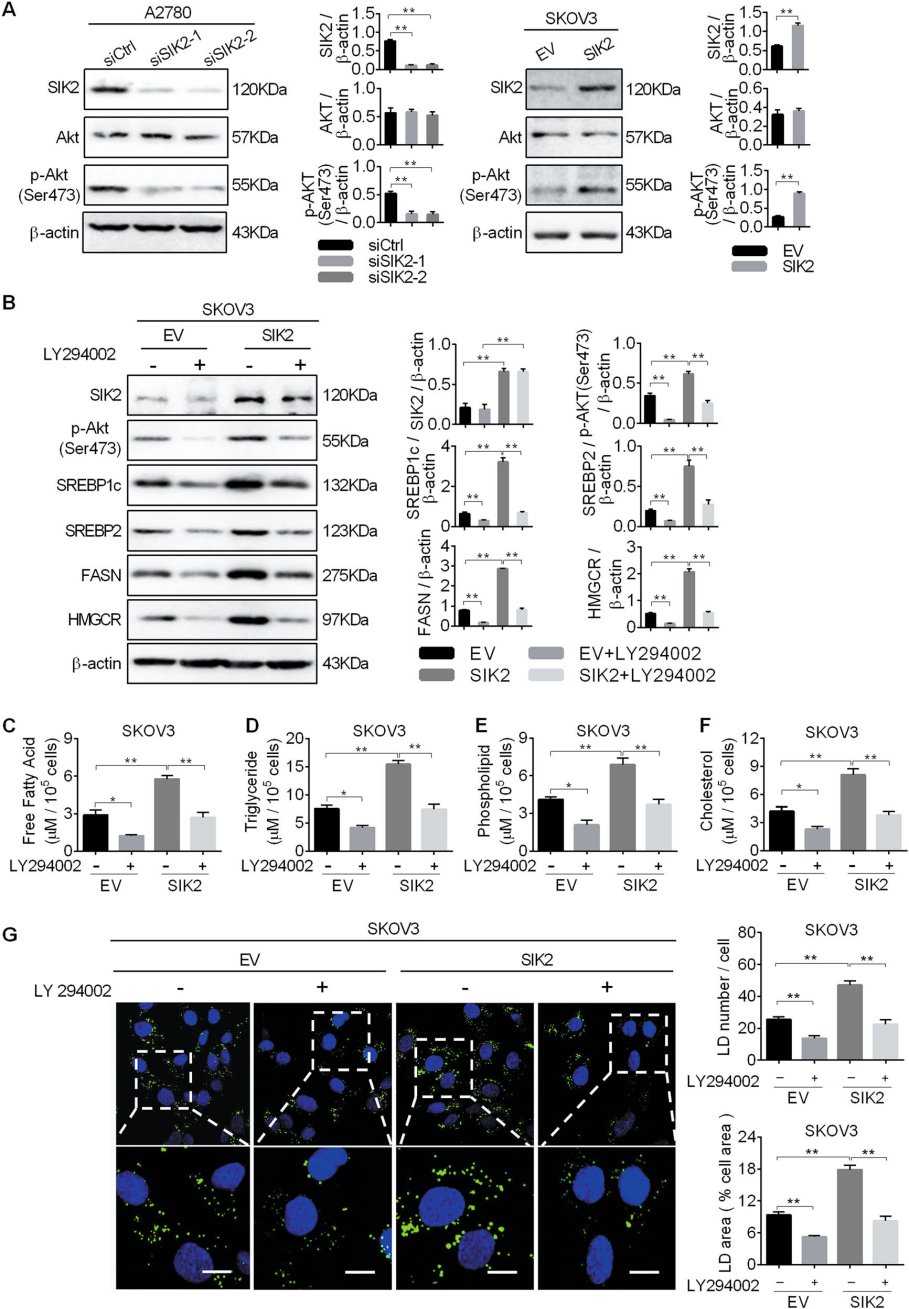
SIK2 promoted the upregulation of SREBP1c and SREBP2 by activating the PI3K/Akt signaling pathway. **a** Western blot analysis for total Akt and phosphorylated Akt (Ser473) in A2780 and SKOV3 cells with treatment as indicated. **b** Western blot analysis for expression of p-Akt (Ser473), SREBP1c, SREBP2, FASN and HMGCR in SKOV3 cells treated with an Akt inhibitor LY294002 (LY294002, 5 μM PI3K/Akt signaling inhibitor for 12 h). **c**–**f** Cellular content of free fatty acid (**c**), triglyceride (**d**), phospholipids (**e**) and cholesterol (**f**) were detected in SKOV3 treated with Akt inhibitor LY294002. **g** The content of neutral lipids were detected by BODIPY 493/503 dye and counterstained with DAPI in SKOV3 cells treated with Akt inhibitor LY294002. Scale bars, 50μm. Average numbers of LDs per cell (upper panel), percentage of cellular area occupied by LDs (lower panel). Data are shown as mean ± S.E.M from three independent experiments. *p < 0.05; **p < 0.01.

Several previous studies have demonstrated that activation of the oncogenic PI3K/Akt pathway plays a critical role in the lipid synthesis^23,24^. Because PI3K/Akt has been reported to be activated by SIK2 in OC cells^17^, we hypothesized that SIK2 activated PI3K/Akt pathway would promote the SREBP1c- and SREBP2-mediated upregulation of fatty acid and cholesterol synthetic enzymes. As shown in Fig. 5a, the phosphorylation level of Akt (Ser473) was significantly decreased in A2780 cells when SIK2 was knocked down, whereas SIK2 overexpression exhibited an opposite effect on the phosphorylation level of Akt (Ser473) in SKOV3 cells. To further study the involvement of PI3K/Akt, we treated OC cells with LY294002, a highly selective PI3K inhibitor. Our results showed that LY294002 treatment markedly decreased the phosphorylation level of Akt (Ser473) in SKOV3 cells with or without SIK2 over-expression. Meanwhile, the expression levels of SREBP1c, SREBP2, FASN and HMGCR were remarkably decreased by LY294002 treatment (Fig. 5b). Expectedly, the decreased levels of intracellular free fatty acid, triglyceride, phospholipids, cholesterol and BODIPY staining of neutral lipids were observed upon treatment with LY294002 (Fig. 5c–g).

### SIK2 promoted the growth of OC cells by enhancing fatty acid and cholesterol synthesis

**Fig. 6:**
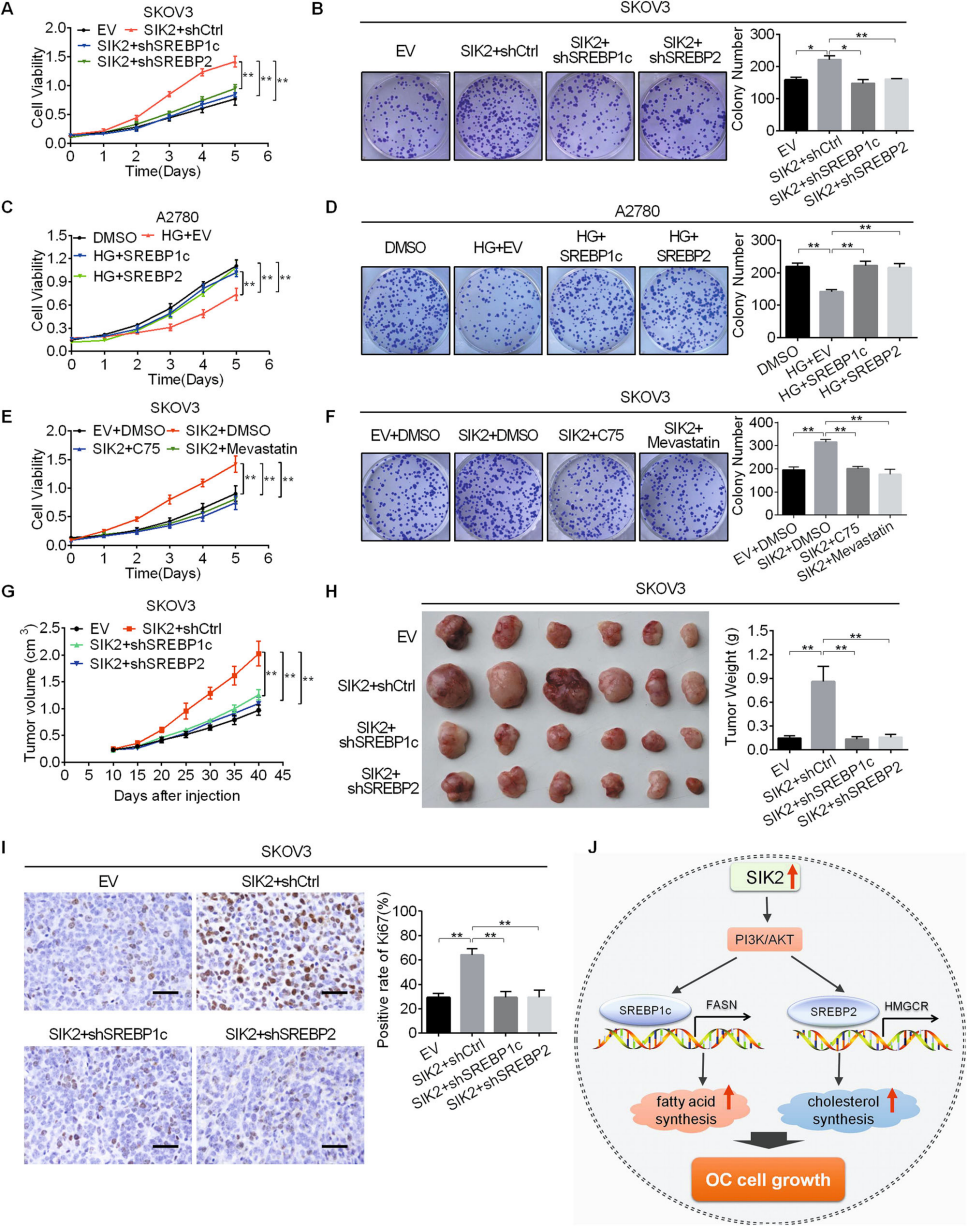
SIK2 promoted the growth of OC cells by enhancing fatty acid and cholesterol synthesis. **a** Cell proliferation ability was evaluated using the MTS assay in SKOV3 cells treated as indicated (EV, empty vector; shCtrl, control shRNA; SIK2, expression vector encoding SIK2; shSREBP1c, shRNAs against SREBP1c; shSREBP2, shRNAs against SREBP2). **b** Colony formation assay in SKOV3 cells treated as indicated. **c** Cell proliferation ability was evaluated using the MTS assay in A2780 cells treated as indicated (HG (SIK inhibitor HG-9-91-01), 4 μM for 8 h; SREBP1c, expression vector encoding SREBP1c; SREBP2, expression vector encoding SREBP2). **d** Colony formation assay in A2780 cells treated as indicated. **e** Cell proliferation ability was evaluated using the MTS assay in SKOV3 cells treated as indicated (EV, empty vector; SIK2, expression vector encoding SIK2; C75, FASN inhibitor, 25 μM for 24 h; Mevastatin, HMGCR inhibitor, 1 μM for 24 h). **f** Colony formation assay in SKOV3 cells treated as indicated. **g** Tumor growth curves of subcutaneous xenograft model for SKOV3 cells with treatment as indicated. **h** The dissected tumors from sacrificed mice and their weight were shown. **i** The expression of Ki-67 in subcutaneous xenografts from different groups were confirmed by immunohistochemistry (IHC) analysis. **j** Schematic depicting the regulation of fatty acid metabolism by SIK2 in OC cells. Scale bars, 50 μm. Data are shown as mean ± S.E.M from three independent experiments. *p *<* 0.05; **p < 0.01.

Considering the important roles of fatty acid and cholesterol synthesis in tumor growth, we hypothesized that SIK2 might promote OC growth through enhancing fatty acid and cholesterol synthesis. To test this hypothesis, pSilencer^TM^3.1-shSREBP1c and pSilencer^TM^3.1-shSREBP2 plasmids were respectively transfected into SKOV3 cells (Supplementary Fig. S3A, B) to construct stable cell lines. We found that SIK2 overexpression significantly promoted the proliferation (Fig. 6a and Supplementary Fig. S3C) and colony formation of SKOV3 cells (Fig. 6b), which were attenuated by either knockdown of SREBP1c or SREBP2. Whereas, overexpression of SREBP1c or SREBP2 restored the inhibitory effects of HG-9-91-01 (an inhibitor of SIK) treatment in A2780 cells (Supplementary Fig. S3D-G and Fig. 6c, d). To provide further supports, fatty acid and cholesterol synthesis were suppressed using C75 (an inhibitor of FASN) and Mevastatin (an inhibitor of HMGCR). As shown in Fig. 6E, F and Supplementary Fig. S3H, SIK2 overexpression significantly promoted the proliferation and colony formation of SKOV3 cells, which were significantly attenuated by treatment with C75^25,26^ at a concentration of 25 μM for 24 h or Mevastatin^27^ at a concentration of 1 μM for 24 h. To evaluate the potential role of enhanced fatty acid and cholesterol synthesis in SIK2-mediated OC growth in vivo, stable cell lines were established and injected subcutaneously into the nude mice. The SIK2 overexpression group exhibited a much faster tumor growth than the control group, whereas the tumor growth-promoting effect of SIK2 was attenuated by knockdown of SREBP1c or SREBP2 (Fig. 6g, h). Immunohistochemical staining analysis indicated a significantly high percentage of Ki-67 positive cells in SIK2-overexpressed xenografts when compared with control, whereas knockdown of SREBP1c or SREBP2 exhibited an opposite effect (Fig. 6i).

Take into account that SIK2 also promotes the metastasis of OC cells^17^, we explored whether SIK2 promoted OC metastasis through enhancing fatty acid and cholesterol synthesis on metastasis of OC cells. As shown in Supplementary Fig. S4A, B, SIK2 overexpression significantly promoted the migration and invasion abilities of OC cells, which were attenuated by either knockdown of SREBP1c or SREBP2. Whereas, overexpression of SREBP1c or SREBP2 restored the effects of HG-9-91-01 treatment in A2780 cells (Supplementary Fig. S4C, D). Expectedly, inhibition of fatty acid and cholesterol synthesis using C75 (inhibitor of FASN) and Mevastatin (inhibitor of HMGCR) showed similar results to the effects of SREBP1c or SREBP2 knockdown (Supplementary Fig. S4E, F). These findings indicate that SIK2 promotes both the growth and metastasis of OC cells through enhancing fatty acid and cholesterol synthesis.

## Discussion

Reprogrammed cellular metabolic pathways of fatty acid and cholesterol synthesis have been recognized as common metabolic hallmarks in many different tumor types, including ovarian cancer^2,28,29^. However, the molecular mechanisms underlying the reprogramming process of lipid metabolism remains largely unknown. In this study, we demonstrated that the SIK2, a member of the AMPK family of kinases, enhanced the fatty acid and cholesterol synthesis in OC cells, which is important for the progression of OC. In addition, our findings indicated that SIK2 enhanced fatty acid and cholesterol synthesis through upregulating the expression of SREBP1c/FASN and SREBP2/HMGCR through activating PI3K/Akt signaling pathway. In contrast, several previous studies in nonmalignant cells such as adipocytes and preadipocytes have demonstrated that SIK2 negatively regulated the lipogenesis^30,31^. These findings suggest that SIK2 may play distinct and even opposite roles in the regulation of lipogenesis in tumor and non-tumor cells, which still needs further confirmation.

The increased de novo fatty acid synthesis is caused by multiple mechanisms, most of which includes the increased expression of lipogenic enzymes. It has been reported that the expression of FASN is increased in high grade serous carcinomas and its overexpression is correlated with poor outcome for women with OC^32,33^. In OC cell lines, overexpression of FASN has also been observed to promote tumor cell growth and its inhibition serves as a promising therapeutic strategy^34,35^. Consistently, our results demonstrated that SIK2 promoted fatty acid synthesis of OC cells through up-regulation of FASN, which further support FASN as a critical oncogene in cancer progression. Compared with most current studies focusing on de novo fatty acid synthesis, cholesterol metabolism in cancer development has received less attention. Currently, in several types of cancer cells, it has been demonstrated that tumor cells display the altered cholesterol metabolism which is characterized by the elevated intracellular cholesterol level. Chushi Li et al. have reported that the rate-limiting enzyme HMGCR for cholesterol synthesis is significantly up-regulated in gastric cancer, which promotes the malignant biological behavior of the cancer cells^11^. Besides, the oncogenic effect of HMGCR has also been demonstrated in glioblastoma and prostate cancer cells^12,36^. In OC cells, HMGCR has been reported to play a role in cisplatin resistance^37^ and its inhibition exhibits anti-metastatic and anti-tumorigenic effects^38^. In accordance, our results demonstrated that SIK2 promoted cholesterol biosynthesis and thus OC cell growth through the up-regulation of HMGCR.

Sterol regulatory element binding proteins (SREBPs) are the most important transcription factors that regulate lipid homeostasis. In mammalian cells, three SREBP isoforms including SREBP1a, SREBP1c, and SREBP2, have been identified. Among the three SREBP isoforms, SREBP1c preferentially activates enzymes involved in fatty acid and triglyceride metabolism, while SREBP2 preferentially activates enzymes involved in cholesterol metabolism^39^. Currently, the activation of both SREBP1c and SREBP2 have been observed in many different types of cancer^18,40–43^, including OC^37,44^. In accordance, our results showed that SREBP1c and SREBP2 expressions and activities were elevated by SIK2 in ovarian cancers. Cumulative evidences have indicated that the expression of SREBPs are tightly regulated by several well-known oncogenic signaling pathways, particularly the PI3K/Akt pathway^23,45,46^, which is frequently activated in human cancer cells^47^. A previous study has demonstrated that PI3K/Akt pathway could be activated by SIK2 in OC cells^17^. Our present study further showed that the activation of the PI3K/Akt signaling was involved in the upregulation of SREBP1c and SREBP2 by SIK2 in OC cells. In addition to SREBPs, ChREBP is another important transcriptional regulator involved in the regulation of lipogenesis. A previous study has demonstrated that SIK2 inhibited the activity of ChREBP transactivation through p300-mediated acetylation in mouse hepatocytes^48^. Accordingly, although no significant effect of SIK2 on the expression of ChREBP were observed in our present study, we still cannot rule out the possibility that ChREBP may also be activated by SIK2 in OC cells and thus play a role in SIK2-regulated lipogenesis and tumor progression.

Increased fatty acid and cholesterol synthesis have been linked to tumorigenesis and cancer progression. Several previous pre-clinical studies have demonstrated that the decrease of cholesterol synthesis inhibits cell proliferation, invasion and metastasis in OC^38,49^. Previous studies have demonstrated that SIK2 plays an important role in the cell proliferation and survival in OC^15,50^. Considering that fatty acid and cholesterol function as the major building blocks and signaling molecules which are required in cancer cells, we propose that the SIK2-regulated fatty acid and cholesterol synthesis may be involved in the OC growth. As expected, our study demonstrated that either over-expression of SREBP1c or SREBP2 robustly reversed the inhibition of growth of OC cells both in vivo and in vitro by SIK2-silencing or inhibition.

In summary, we demonstrate a key regulatory role of SIK2 in lipid metabolism of OC cells. Our study provides novel insights to understand the molecular mechanisms underlying the reprogrammed lipid metabolism in cancer cells, as well as a strong line of evidence for this molecule to be used a therapeutic target in OC treatment.

## Supplementary information

SUPPLEMENTAL MATERIAL

SUPPLEMENTAL MATERIAL

SUPPLEMENTAL MATERIAL

SUPPLEMENTAL MATERIAL

SUPPLEMENTAL MATERIAL

SUPPLEMENTAL MATERIAL

SUPPLEMENTAL MATERIAL
