# Supplementary figures and images for: SIK2 enhances synthesis of fatty acid and cholesterol in ovarian cancer cells and tumor growth through PI3K/Akt signaling pathway
Source: Cell Death Dis. 2020 Jan 13;11(1):25. doi: 10.1038/s41419-019-2221-x (PMC6957524; doi:10.1038/s41419-019-2221-x)

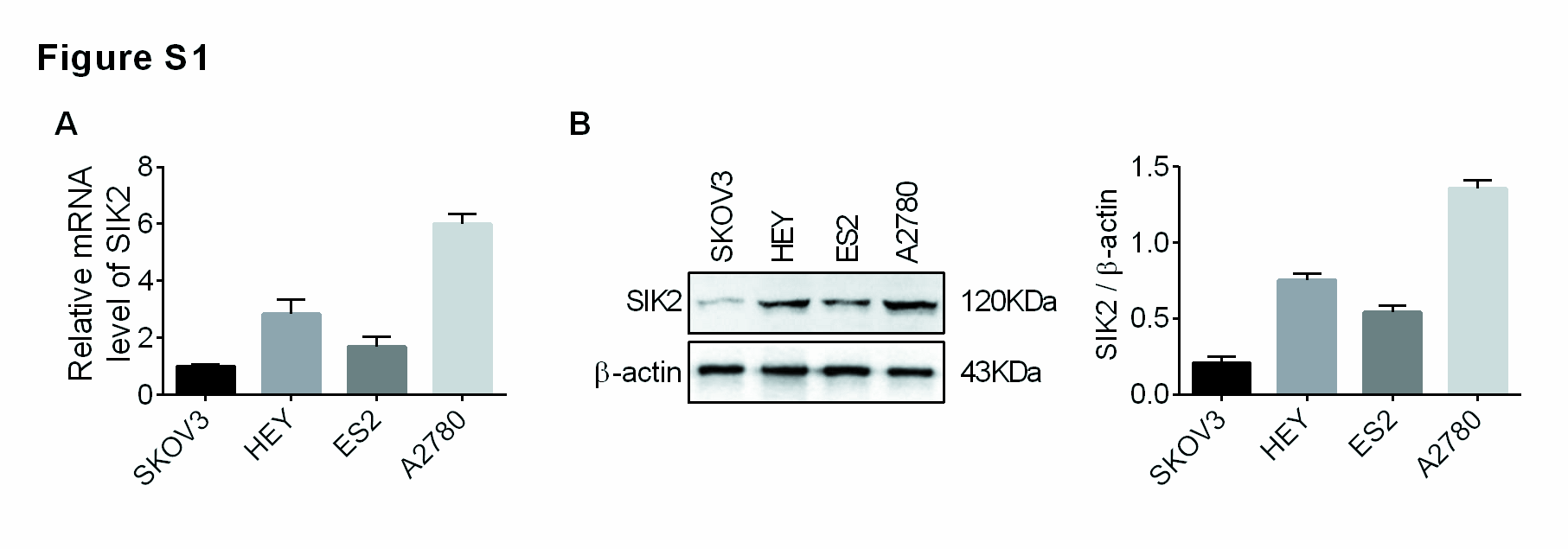

Supplement: Supplementary file 1 — SUPPLEMENTAL MATERIAL [file 41419_2019_2221_MOESM1_ESM.tif]

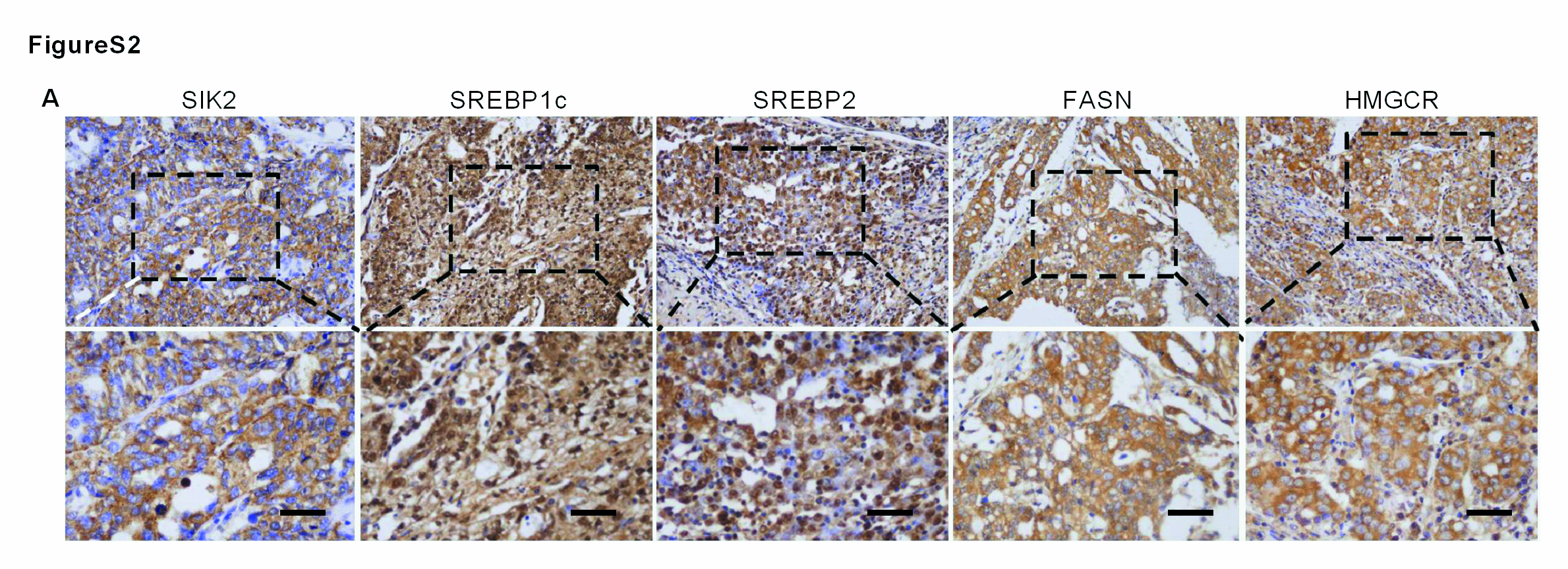

Supplement: Supplementary file 2 — SUPPLEMENTAL MATERIAL [file 41419_2019_2221_MOESM2_ESM.tif]

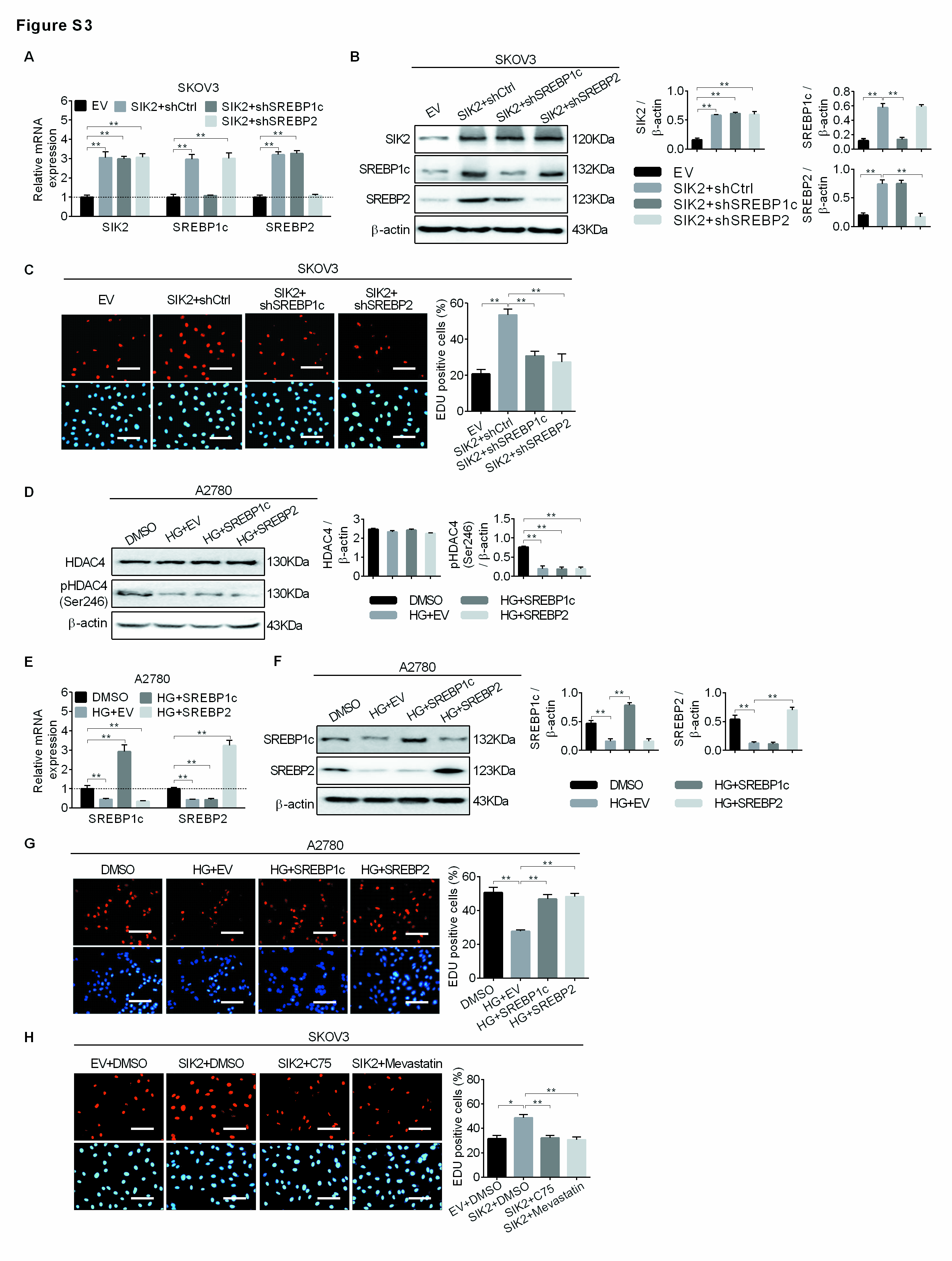

Supplement: Supplementary file 3 — SUPPLEMENTAL MATERIAL [file 41419_2019_2221_MOESM3_ESM.tif]

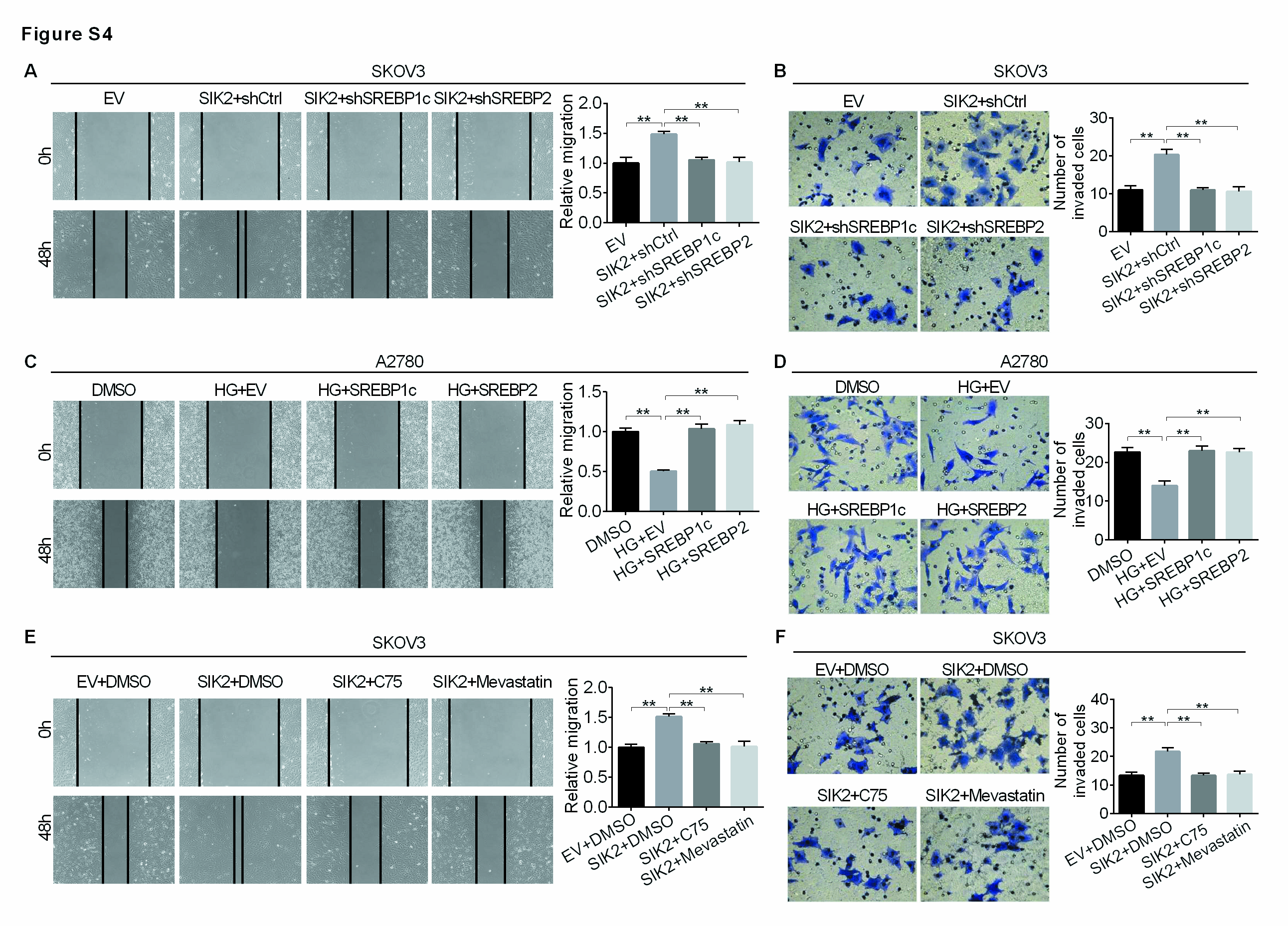

Supplement: Supplementary file 4 — SUPPLEMENTAL MATERIAL [file 41419_2019_2221_MOESM4_ESM.tif]
