# Supplementary material for: SIK2 enhances synthesis of fatty acid and cholesterol in ovarian cancer cells and tumor growth through PI3K/Akt signaling pathway
Source: Cell Death Dis. 2020 Jan 13;11(1):25. doi: 10.1038/s41419-019-2221-x (PMC6957524; doi:10.1038/s41419-019-2221-x)
Supplement: Supplementary file 5 — SUPPLEMENTAL MATERIAL [file 41419_2019_2221_MOESM5_ESM.docx]

**Supplementary Figure Legends**

**SIK2 enhances synthesis of fatty acid and cholesterol in ovarian cancer cells and tumor growth through PI3K/Akt signaling pathway**

**Figure S1.** Quantitative RT-PCR (A) and Western blot (B) analyses for the expression levels of SIK2, SREBP1c and SREBP2 were in SKOV3 cells treated as indicated (EV, empty vector.; shCtrl, control shRNA; SIK2, expression vector encoding SIK2; shSREBP1c, shRNAs against SREBP1c; shSREBP2, shRNAs against SREBP2)

**Figure S2.** Representative immunohistochemical staining images of SIK2, SREBP1c, SREBP2, FASN and HMGCR in tumor tissue sections from OC patient (Scale bars, 100 μm).

**Figure S3. SIK2 promoted the upregulation of SREBP1c and SREBP2 by activating the Akt/mTOR signaling pathway.** Quantitative RT-PCR (A) and Western blot (B) analyses for the expression levels of SIK2, SREBP1c and SREBP2 in SKOV3 cells treated as indicated (EV, empty vector.; shCtrl, control shRNA; SIK2, expression vector encoding SIK2; shSREBP1c, shRNAs against SREBP1c; shSREBP2, shRNAs against SREBP2). (C) EdU cell proliferation assay was applied in SKOV3 cells with treatment as indicated. (D) The efficiency of SIK2 inhibition by HG-9-91-01 were determined by Western blot analysis of the level of pHDAC4 (Ser246). (HG, SIK inhibitor HG-9-91-01; SREBP1c, expression vector encoding SREBP1c; SREBP2, expression vector encoding SREBP2). Quantitative RT-PCR (E) and Western blot (F) analyses for the expression levels of SREBP1c and SREBP2 were in A2780 cells treated as indicated (HG, SIK inhibitor HG-9-91-01; SREBP1c, expression vector encoding SREBP1c; SREBP2, expression vector encoding SREBP2). (G) EdU cell proliferation assay was applied in A2780 cells with treatment as indicated. (H) EdU cell proliferation assay was applied in SKOV3 cells with treatment as indicated (EV, empty vector.; SIK2, expression vector encoding SIK2; C75, FASN inhibitor, 25 μM C75 for 24 h; Mevastatin, HMGCR inhibitor, 1 μM Mevastatin for 24 h). ( Data are shown as mean ± S.E.M from three independent experiments. *p <0.05; **p <0.01.)

**Figure S4. SIK2 promoted the the migration and invasion abilities of OC cells by enhancing fatty acid and cholesterol synthesis in vitro.**

(A and B) Cell migration and invasion were evaluated by the scratch wound healing (A), transwell matrigel invasion (B) assays in SKOV3 cells with treatments as indicated (EV, empty vector; SIK2, expression vector encoding SIK2; shCtrl, control shRNA; SIK2, expression vector encoding SIK2; shSREBP1c, shRNAs against SREBP1c; shSREBP2, shRNAs against SREBP2). (C and D) Cell migration and invasion were evaluated by the scratch wound healing (C) and transwell matrigel invasion (D) assays in SKOV3 cells with treatments as indicated (HG, SIK inhibitor HG-9-91-01; SREBP1c, expression vector encoding SREBP1c; SREBP2, expression vector encoding SREBP2). (E and F) Cell migration and invasion were evaluated by the scratch wound healing (E) and transwell matrigel invasion (F) assays in A2780 cells with treatments as indicated (EV, empty vector; SIK2, expression vector encoding SIK2; C75, FASN inhibitor 25 μM C75 for 24 h; Mevastatin, HMGCR inhibitor 1 μM Mevastatin for 24 h). Data are shown as mean ± S.E.M from three independent experiments. *p <0.05; **p <0.01.
