# Supplementary material for: SIK2 enhances synthesis of fatty acid and cholesterol in ovarian cancer cells and tumor growth through PI3K/Akt signaling pathway
Source: Cell Death Dis. 2020 Jan 13;11(1):25. doi: 10.1038/s41419-019-2221-x (PMC6957524; doi:10.1038/s41419-019-2221-x)
Supplement: Supplementary file 6 — SUPPLEMENTAL MATERIAL [file 41419_2019_2221_MOESM6_ESM.docx]

**Supplementary Table S1.**

Primary antibodies used for western blot and immunohistochemistry.

| **Antibody** | **Company (Cat. No.)** | **Working Concentration Dilutions** |
| --- | --- | --- |
| SIK2 | Abcam (ab115567 ) | WB: 1/1000 IHC:1/200 |
| ACC1 | Abcam (ab45174) | WB: 1/1000 |
| SCD1 | Abcam (ab19862) | WB: 1/800 |
| ACLY | Abcam (ab40793) | WB: 1/1000 |
| FASN | Abcam (ab128870) | WB: 1/1000 |
| HMGCR | Therm Fisher (MA5-31336) | WB: 1/1000 |
| HMGCS1 | Proteintech (17643-1-AP) | WB: 1/500 |
| chREBP | Proteintech (13256-1-AP) | WB: 1/1000 |
| SREBP1c | Proteintech (66875-1-Ig) | WB: 1/1000 |
| SREBP2 | Abcam (ab30682) | WB: 1/500 |
| Lamin B1 | Abcam(ab65986) | WB: 1/1000 |
| Ki-67 | Proteintech (27309-1-AP) | IHC:1/200 |
| Akt | Cell Signaling (#9272S) | WB:1/1000 |
| p-Akt (Ser473) | Cell Signaling (#4051S) | WB:1/1000 |
| VDAC | Proteintech (10866-1-AP) | WB:1/1000 |
| β-actin | Beijing TDY (TDY051F) | WB: 1/3000 |
| HDAC4 | Abcam (ab12172) | WB: 1/800 |
| p-HDAC4 (Ser246) | Abcam (ab39408) | WB: 1/500 |
