# Supplementary material for: SIK2 enhances synthesis of fatty acid and cholesterol in ovarian cancer cells and tumor growth through PI3K/Akt signaling pathway
Source: Cell Death Dis. 2020 Jan 13;11(1):25. doi: 10.1038/s41419-019-2221-x (PMC6957524; doi:10.1038/s41419-019-2221-x)
Supplement: Supplementary file 7 — SUPPLEMENTAL MATERIAL [file 41419_2019_2221_MOESM7_ESM.docx]

**Supplementary Table S2.**

Sequence of primers for qRT-PCR analysis

| 1. Primers used in q-PCR analysis | | | | | |
| --- | --- | --- | --- | --- | --- |
| **Gene** | | **Forward Primer** | | **Reverse Primer** | |
| SIK2 | | CAGCAGCTGCAGGAACATAG | | GACTTGGCTGTGGGTAGGAG | |
| ACC1 | | AGGAAGATGGTGTCCGCTCTG | | GGGGAGATGTGCTGGGTCAT | |
| SCD1 | | CCCCACCTACAAGGATAAGGA | | CACGAGCCCATTCATAGACAT | |
| ACLY | | CAGCAGGACAGCATCTTTTTC | | TGGACTTGGGACTGAATCTTG | |
| FASN | | CTTGGGTGCCGATTACAACC | | GCCCTCCCGTACACTCACTC | |
| HMGCR | | TGATTGACCTTTCCAGAGCAAG | | CTAAAATTGCCATTCCACGAGC | |
| HMGCS1 | | GATGTGGGAATTGTTGCCCTT | | ATTGTCTCTGTTCCAACTTCCAG | |
| chREBP | | GAAGCCACCCTATAGCTCCC | | GAAGCCACCCTATAGCTCCC | |
| SREBP1c | | GGAGCCATGGATTGCACATT | | CAGGAAGGCTTCCAGAGAGG | |
| SREBP2 | | GCGTTCTGGAGACCATGGA | | ACAAAGTTGCTCTGAAAACAAATCA | |
| β-actin | | TCGCCTTTGCCGATCCG | | ATGATCTGGGTCATCTTCTCG | |
| **2. siRNAs** | | | | | |
| **Gene** | | | **Forward** | | **Reverse** |
| siSIK2-1 | | | GGACCGACUCUUCCAAUUU | | AAAUUGGAAGAGUCGGUCCTT |
| siSIK2-2 | | | GGUGUGUGCUAUUGCAUAU | | AUAUGCAAUAGCACACACCTT |
| siSREBP1c | | | GGAGGCUUCUCUACAGGAATT | | UUCCUGUAGAGAAGCCUCCTT |
| siSREBP2 | | | GCGCUCUCAUUUUACCAAATT | | GGAUGAUGCCAAAGGUCAAATT |
| siControl | | | UUCUCCGAACGUGUCACGUTT | | ACGUGACACGUUCGGAGAATT |
